# COX2 expression is associated with preoperative tumor volume but not with volumetric tumor growth in vestibular schwannoma

**DOI:** 10.1186/s42466-021-00111-6

**Published:** 2021-03-01

**Authors:** Felix Behling, Elisa Suhm, Vanessa Ries, Vítor Moura Gonçalves, Ghazaleh Tabatabai, Marcos Tatagiba, Jens Schittenhelm

**Affiliations:** 1 Department of Neurosurgery, University Hospital Tübingen, Eberhard-Karls-University Tübingen, Hoppe-Seyler Street 3, Tübingen, Germany; 2 Center for Neuro-Oncology, Comprehensive Cancer Center Tübingen – Stuttgart, University Hospital Tübingen, Eberhard-Karls-University Tübingen, Tübingen, Germany; 3 Faculty of Medicine, University of Porto, 4200-319 Porto, Portugal; 4 Department of Neurology and Interdisciplinary Neuro-Oncology, University Hospital Tübingen, Eberhard-Karls-University Tübingen, Tübingen, Germany; 5 Hertie Institute for Clinical Brain Research, Tübingen, Germany; 6 German Cancer Consortium (DKTK), DKFZ partner site Tübingen, Tübingen, Germany; 7 Department of Neuropathology, University Hospital Tübingen, Eberhard-Karls-University Tübingen, Tübingen, Germany

**Keywords:** Vestibular schwannoma, Acoustic neuroma, COX2, Tissue microarray, MIB1, Volumetric tumor growth

## Abstract

**Objective:**

Vestibular schwannomas (VS) are benign slow growing tumors arising from the vestibular nerve. The role of cyclooxygenase 2 (COX2) in tumor development of growth has been addressed in a few studies with contradictory results and suggestions. We recently analyzed the immunohistochemical expression of COX2 in 1044 VS samples and described an association of higher COX2 expression with proliferation but found no influence by regular intake of acetylsalicylic acid. We now collected volumetric radiographic data of the preoperative tumor volume and growth to further test the role of COX2 in VS growth.

**Methods:**

Preoperative images of 898 primary sporadic vestibular schwannomas were assessed, and sufficient preoperative imaging was used for the volumetric measurement preoperative tumor volume (*n* = 747) and preoperative relative tumor growth (*n* = 171). Clinical parameters and results of the immunohistochemical expression of COX2 and MIB1 in resected tumor tissue samples were obtained from our prior study. ANOVA, CART-analysis and multivariate nominal logistic regression were used for statistical analysis.

**Results:**

Larger preoperative tumor volumes were observed with tumors of younger patients (*p* = 0.0288) and with higher COX2 expression scores (*p* < 0.0001). Higher MIB1 expression was associated with smaller tumors (*p* = 0.0149) but with increased radiographic tumor growth (*p* = 0.0003). Patients of older age had tumors with slower growth rates (*p* = 0.0311). In the multivariate analysis only MIB1 expression was an independent significant factor regarding tumor growth (*p* = 0.0002).

**Conclusions:**

Higher expression of COX2 in schwannoma is associated with an increased preoperative tumor volume but not with radiographic tumor growth over time.

## Background

Vestibular schwannoma (VS) is the most common intracranial nerve sheath tumor accounting for approximately 6% of primary intracranial neoplasms overall [16]. This benign tumor is typically encapsulated and consists of well-differentiated schwann cells. Morphological variants include cellular, plexiform and melanotic schwannoma. Approximately 90% are sporadic, while multiple schwannomas and hybrid schwannoma/neurofibroma nerve sheath tumors are overrepresented in neurofibromatosis type 2 (NF2) and schwannomatosis [8]. While microsurgical resection or radiosurgery are effective treatments, recurrent tumors are difficult to treat, especially due to the lack of alternative therapy options. Furthermore, VSs with cystic aspects are more challenging to manage as well [15]. Besides bevacizumab, which has been explored for the stabilization of VS in patients suffering from neurofibromatosis type 2 (NF2) with unconvincing results [14, 18], there are no established treatment alternatives.

During the last years, inflammatory processes and the tumor microenvironment have been subject to a growing research effort in oncology which also extended into the field of vestibular schwannoma [4, 7, 9]. One of the subjects of extensive research is the enzyme cyclooxygenase 2 (COX2) with newly gained insights in several cancers [13, 22] including vestibular schwannoma [5, 10]. A decisive role in tumor development and progression has been advocated. Tumor proliferation and increased cell motility by COX2 have been described, as well as inhibition of apoptosis [3]. Increased expression levels have been reported in the most common tumor types such as cancer of the colon, lung, breast and prostate. Furthermore, the selective pharmacological inhibition by celecoxib is possible and first promising results have been shown [20].

A recent study by our research group demonstrated a significant association of immunohistochemical detection of COX2 and proliferative marker expression (MIB1) in a large cohort of vestibular schwannomas [1]. We have now conducted volumetric tumor measurements of preoperative tumor size and growth in this cohort to further examine the possible proliferative effect of COX2 and proliferation marker MIB1 in VS.

## Materials and methods

### Tübingen Schwannoma patient cohort and data

Patients that were surgically treated in our institution between October 2003 and May 2017 for primary sporadic vestibular schwannoma were evaluated for inclusion in this retrospective single center study (*n* = 979). Cases without consent for scientific tissue analysis (*n* = 26), missing clinical data (*n* = 16), unsuitable tumor tissue (*n* = 14), prior radiotherapy (*n* = 20) and lack of sufficient imaging data (*n* = 156) were excluded, leaving 747 vestibular schwannomas for analysis. Adequate serial imaging for radiographic growth calculation (see below) was available for 171 cases. Clinical data was collected via review of electronic patient charts.

### Radiographic volumetric tumor measurement

Preoperative magnetic resonance imaging (MRI) data was used for radiographic tumor volumetry using the Brainlab iPlan Net software 2.4 (Brainlab AG, Munich, Germany). For a few cases sufficient contrast-enhanced computer tomographies (CT) were also used for volumetric measurements. Images that were done more than 6 months prior to surgery were considered. Growth analysis was only included if images with appropriate quality for sufficient volumetry were available with at least an interval of 3 months in between. Tumor volume was measured in cm^3^ and growth was calculated as percentual increase in volume per year.

### Immunohistochemistry

Available data from immunohistochemical staining for COX2 and MIB1 according to our prior study were used [1]. Tissue microarrays (TMA) were constructed with 2 representative 1 mm tissue probes for each tumor using a conventional microarrayer (Beecher Instruments, Sun Prairie, Wisconsin, USA). With microtomy 4 μm slices were produced and dried at 80° for 15 min. A Ventana BenchMark immunostainer (Ventana Medical Systems, Tucson, Arizona, USA) was used for immunohistochemical staining of COX2 (1:800, Biozol, Eching, Germany). Quantification was done by semiquantitative assessment and formulation of an intensity distribution score (ID-score): 0 = 0–5%, 1 = 5–25%, 2 = 25–50%, 3 = 50–75%, 4 = 75–100%). MIB1 expression was analyzed via quantification on digitalized whole section stainings that were done within the routine neuropathological workup.

### Statistical methods

For statistical analysis JMP® Statistical Discovery Software, version 15.1.0 (Cary, NC: SAS Institute Inc.; 1989) was used. ANOVA and multivariate regression analysis were applied with a significance level of α < 0.05. Classification and regression tree analysis (CART) was done to define optimal dichotomous cut offs for the variables age and the expression of MIB1 and COX2. The study was approved by the Clinical Ethics Committee of the University of Tübingen (Project number: 336/2017BO2).

## Results

### Study cohort characteristics

Overall, 747 patients diagnosed with a primary sporadic vestibular schwannoma and available radiologic data were included in the study with 380 (50.9%) of female and 367 (49.1%) of male gender. The mean age was 48.9 years, ranging from 7.0 to 80.1 years.

### Immunohistochemical expression of MIB1 and COX2 indicates low proliferative activity

These results present part of the data recently published [1]. Digital analysis with quantification measurement was done in 733 cases. In 14 cases MIB1 staining was not suitable for digital analysis. The mean MIB1 immunopositivity was 1.34% with a range from 0 to 4.75%.

**Fig. 1:**
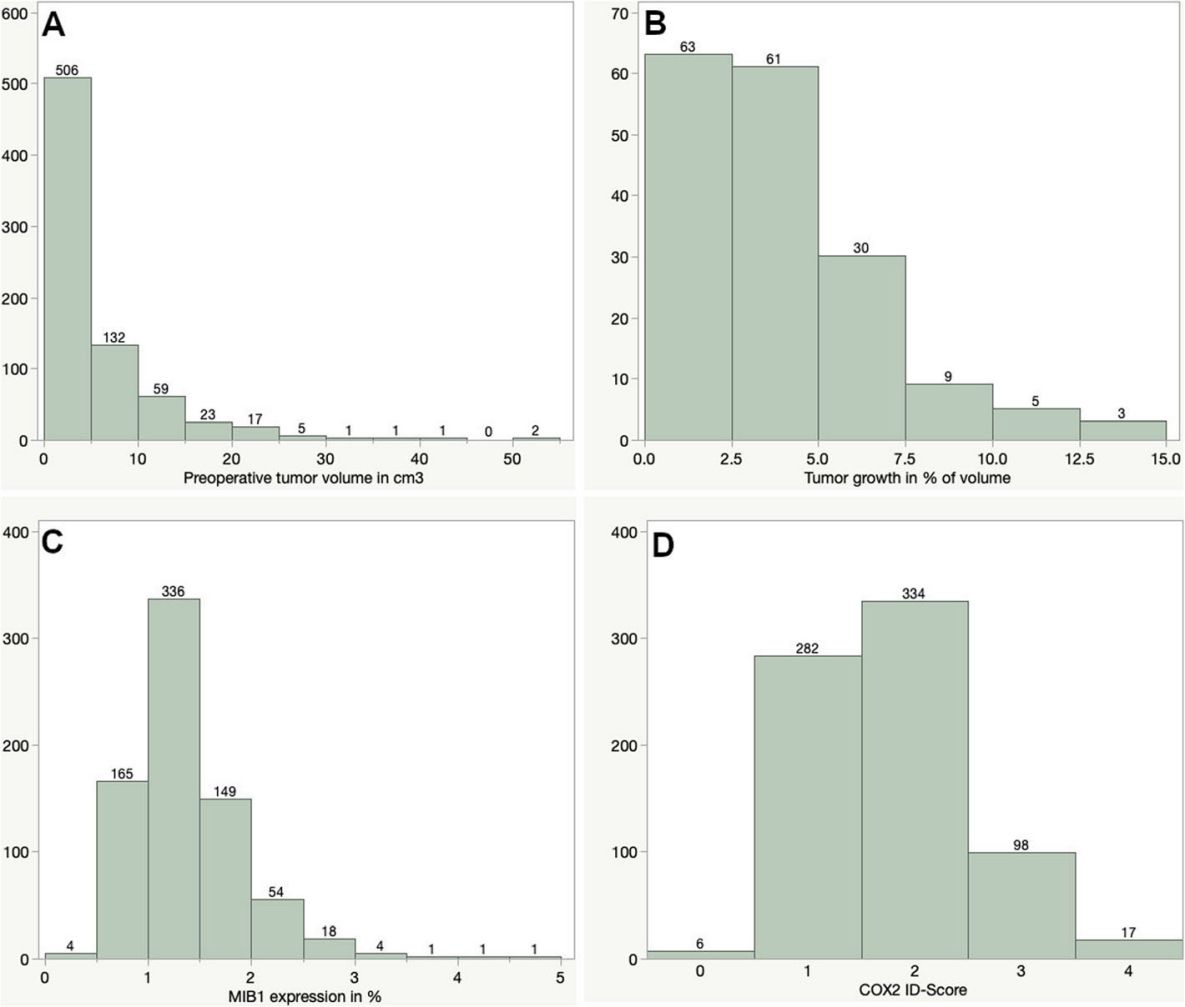
Preoperative tumor volume according to gender (**a**), age (**b**), MIB1 (**c**) and COX2 expression (D and E) (ANOVA, asterisk represents statistically significant results)

COX2 expression was assessable for 737 tumors, while staining was insufficient for further analysis in 10 cases. Only 6 cases were graded as negative and the majority of tumors reached an ID-score of 1 or 2 (*n* = 282 (38.3%) and 334 (45.3%), respectively). A score of 3 and 4 was reached by 98 (13.3%) and 17 (2.3%). A more detailed illustration of the distribution of MIB1 and COX2 immunohistochemical expression can be seen in Fig. 1.

### Larger radiographic tumor volumetry is associated with low proliferation

**Fig. 2:**
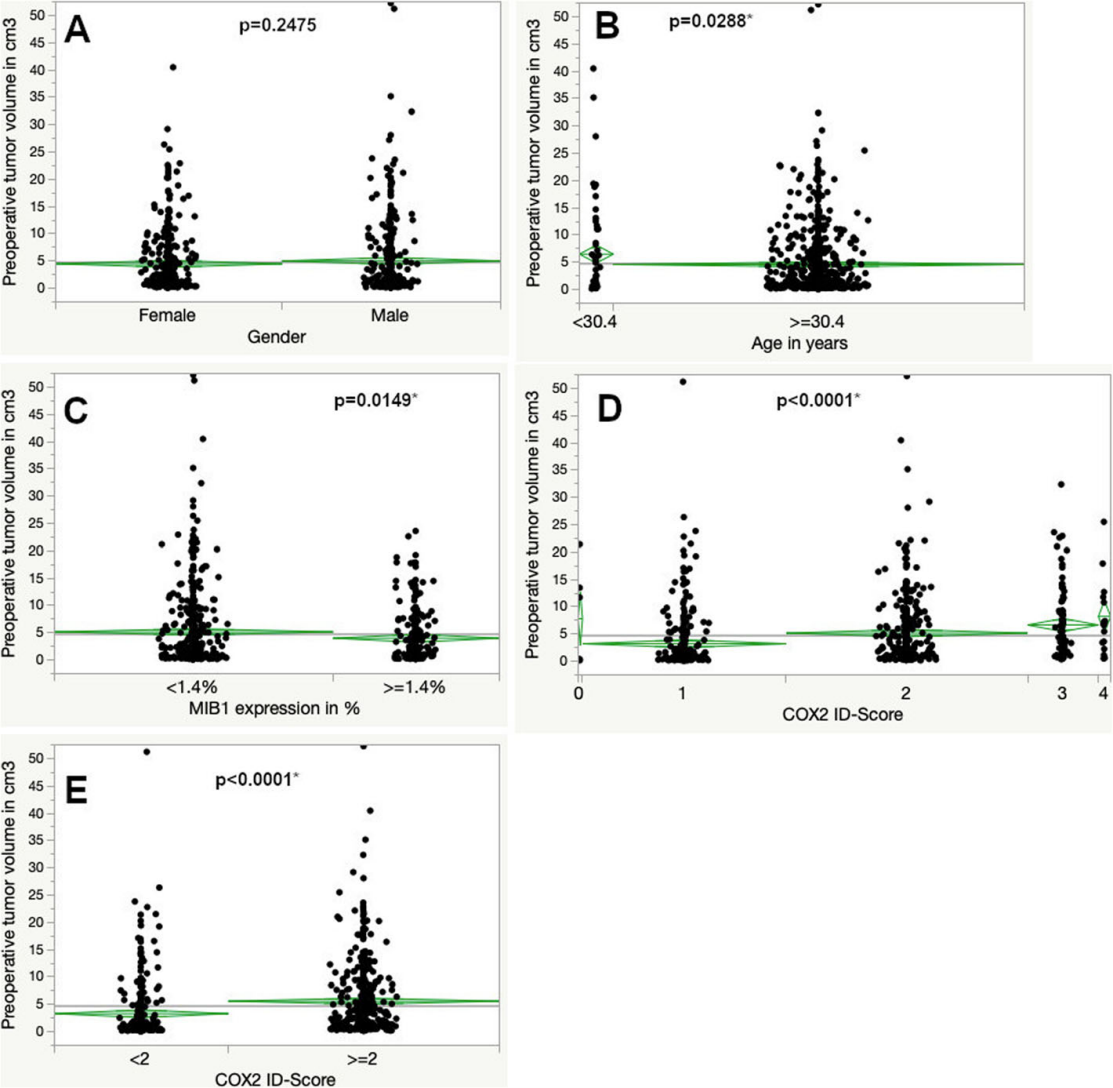
Tumor growth in percent increase of volume per year according to gender (**a**), age (**b**), MIB1 (**c**) and COX2 expression (**d** and **e**) (ANOVA, asterisk represents statistically significant results)

The mean tumor volume was 4.75 cm^3^, ranging from 0.04 to 52.1 cm^3^. There was no difference in preoperative tumor volume regarding gender. Patients younger than 30.4 years had a mean preoperative tumor volume of 6.50 cm^3^ compared to 4.60 cm^3^ of older patients (*p* = 0.0288). Cases with a MIB1 expression < 1.4% had a larger tumor volume than tumors with a proliferative marker expression of 1.4% or higher (5.16 compared to 3.99 cm^3^, *p* = 0.0149). Cases with increased COX2 ID-scores showed higher preoperative tumor volumes (*p* < 0.0001, see Fig. 2, Table 1).

**Table 1:** Differences in preoperative volumetry

	N (%)	Mean tumor volume in cm^3^	*p*-value (ANOVA)
Gender			
Female	380 (50.9)	4.49	0.2475
Male	367 (49.1)	5.02	
Age			
< 30.4 years	57 (7.6)	6.50	0.0288*
> =30.4 years	690 (92.4)	4.60	
MIB1			
< 1.4%	459 (62.6)	5.16	0.0149*
> =1.4%	274 (37.4)	3.99	
COX2 expression			
negative	6 (0.8)	7.80	< 0.0001*
1	282 (38.3)	3.22	
2	334 (45.3)	5.16	
3	98 (13.3)	6.65	
4	17 (2.3)	8.22	
< 2	288 (39.1)	3.31	
> =2	449 (60.9)	5.60	< 0.0001*

Asterisk (*) represents statistically significant results

**Fig. 3:**
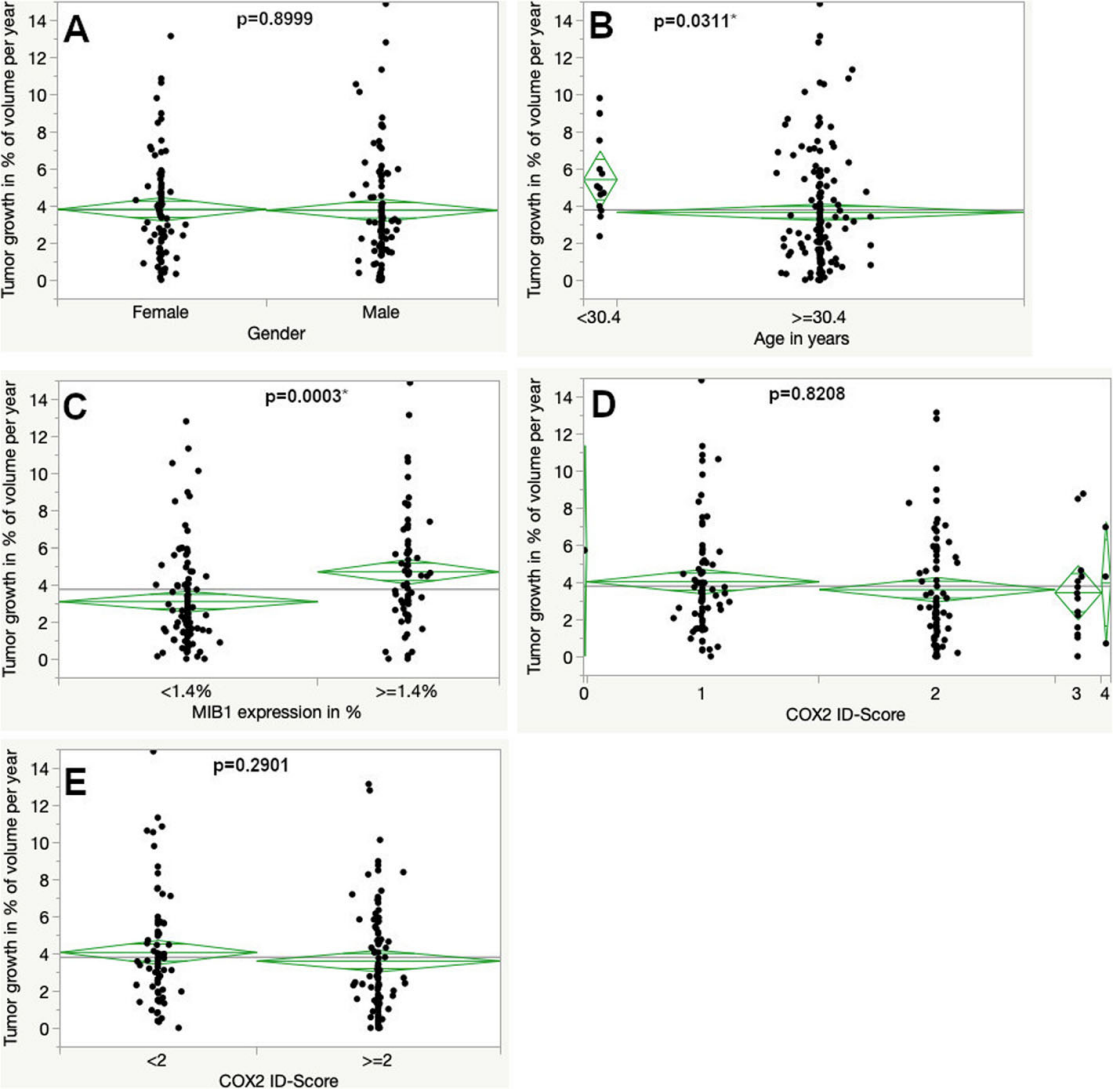
Distribution of preoperative tumor volume (**a**), percentual tumor growth (**b**), MIB1 expression (**c**) and COX2 ID-score (**d**). ANOVA, asterisk (*) represents statistically significant results

Volumetric growth analysis was available for 171 cases (22.9%). The mean tumor growth was 3.81%/year with the most pronounced growth rate at 14.87%/year. There was no difference in growth rate regarding gender or COX2 expression. Younger patients showed faster tumor growth compared to patients 30.4 years or older (5.44 vs. 3.67%/year, *p* = 0.0311). Cases with a MIB1 expression reaching or surpassing 1.4% showed a mean increase in volume of 4.72% compared to tumors with a lower expression rate (3.12%/year, *p* = 0.0003). Details are displayed in Fig. 3 and Table 2.

**Table 2:** Differences in volumetric growth

	N (%)	%growth/year	*p*-value (ANOVA)
Gender			
Female	81 (47.4)	3.84	0.8999
Male	90 (52.6)	3.78	
Age			
< 30.4 years	13 (7.6)	5.44	0.0311*
> =30.4 years	158 (92.4)	3.67	
MIB1			
< 1.4%	97 (58.8)	3.12	0.0003*
> =1.4%	68 (41.2)	4.72	
COX2 expression			
negative	1 (0.6)	5.71	0.8208
1	75 (44.1)	4.06	
2	76 (44.7)	3.63	
3	15 (8.8)	3.46	
4	3 (1.8)	3.98	
< 2	76 (44.7)	4.08	0.2901
> =2	94 (55.3)	3.61	

Asterisk (*) represents statistically significant results

### Multivariate analysis identifies MIB1 but not COX2 expression to be associated with radiographic tumor growth

Nominal logistic regression of all relevant markers was done for multivariate assessment (*n* = 165). Only a MIB1 expression equal or exceeding 1.4% was revealed as an independent marker for radiographic vestibular schwannoma growth (*p* = 0.0002). COX2 ID-score below 2 missed statistical significance (*p* = 0.1006). Age and gender had also no independent association with volumetric tumor growth (Table 3).

**Table 3:** Results of the nominal logistic regression of percentual volumetric tumor growth

	Estimate	Std Error	t Ratio	Lower 95%	Upper 95%	*p*-value
Intercept	4.53	0.41	10.97	3.71	5.35	< 0.0001*
Gender (Female)	0.14	0.22	0.63	−0.29	0.57	0.5302
Age (< 30 years)	0.66	0.41	1.58	−0.16	1.47	0.1153
MIB1 (< 1.4%)	−0.84	0.22	−3.76	−1.28	−0.40	0.0002*
COX2 (< 2)	0.36	0.22	1.65	−0.07	0.79	0.1006

Asterisk (*) represents statistically significant results

## Discussion

The role of inflammatory-mediated markers, especially COX2 has been assessed in different tumor types and a decisive role in cancer development and progression has been suggested [13, 22]. First insights were also gained in vestibular schwannoma [10] [5]. Several retrospective analyses of radiographic tumor growth and aspirin intake were conducted with contradictory results [11, 12], which led to a prospective clinical trial that is currently recruiting patients with vestibular schwannoma under observation for pharmacological treatment with aspirin or placebo for radiographic growth control (NTC03079999).

Overall, the role of COX2 in vestibular schwannoma growth is far from being clearly understood. We therefore previously performed a retrospective tissue analysis of the immunohistochemical expression of COX2 and MIB1 in 1048 vestibular schwannoma [1]. We described clinical subgroups with different COX2 expression and showed that a higher rate of MIB1 expression was seen in tumors with higher COX2 expression, suggesting a possible association of COX2 and proliferation. Although the difference in MIB1 was statistically significant, the overall expression of this proliferation marker is notoriously low in a slow growing tumor like vestibular schwannoma. Therefore, it is questionable if the difference in MIB1 expression alone among the different COX2 expression groups is of biological relevance regarding tumor growth. To further address this issue, we have done volumetric measurements of preoperative radiographic images to assess the preoperative tumor volume and growth rate in order to verify the proliferative impact of COX2 positive cells and MIB1 expression in vestibular schwannoma.

The results of this study show that besides the significant increase of MIB1 expression with higher COX2 ID-score [1], there is no significant difference in radiographic tumor growth when regarded as percentual change in tumor volume. Our data indicate that COX2-mediated inflammatory processes in vestibular schwannoma do not influence tumor growth. Thus, the recommendation to control tumor growth conservatively with acetyl salicylic acid via COX2 inhibition [21] cannot be supported. On the other hand, inflammatory infiltration and COX2 expression could also be an expression of reactive changes in the tumor tissue, maybe even based on an immune response towards schwannoma cells that does not necessarily have to be associated with tumor growth.

Regarding the preoperative tumor volume, relative higher COX2 expression is associated with larger vestibular schwannoma volume, which was already suggested in our prior study where a correlation of COX2 expression and the tumor extension (Hannover classification) was described [1]. An association of immune cell infiltration and vestibular schwannoma size has also been recently described [2] and could be a possible explanation for this finding. A result that is harder to grasp, is the preoperative tumor volume according to our MIB1 expression results. Surprisingly, tumors with increased nuclear expression for this proliferation marker had a smaller preoperative volume in contrast to our COX2 data. At this point we do not have a clear explanation for this unexpected result, but it can be hypothesized that the growth dynamics in vestibular schwannoma may be more complex due to the different surrounding tissue during tumor growth. Vestibular schwannomas first encounter the bony limits of the internal auditory canal and with further progression enter the wide cerebellopontine cistern where less resistance is encountered until the cerebellar peduncle and brainstem are reached. Widening of the internal auditory canal is regularly observed in vestibular schwannomas and a correlation with tumor consistency has been suggested [19]. Whether a dynamic in growth rate in interaction with the surrounding structures exists has not yet been investigated but cases with more extensive invasive growth have been described [17]. In malignant tumors, a frequent epigenetic shift between proliferative and invasive phenotypes or simply expressing both phenotypes simultaneously is common [6]. To our knowledge, this concept has not yet been investigated in vestibular schwannomas.

The main limitation of the study is its retrospective nature. Only cases with complete datasets were considered for multivariate radiographic growth analysis. Sufficient preoperative imaging with appropriate time intervals and imaging quality was only available for a subset of cases, limiting the multivariate analysis to 165 of the 747 vestibular schwannomas. The majority of vestibular schwannomas treated in our center shows a larger tumor extent and therefore underwent microsurgical resection without further follow-up imaging.

## Conclusion

The expression of COX2 in vestibular schwannoma is associated with the preoperative tumor volume and the expression of the proliferative marker MIB1 but not with radiographic tumor growth.

## Data Availability

The dataset of the study is available from the corresponding author on reasonable request.

## Abbreviations

ANOVA: Analysis of variance
CART: Classification and regression tree
COX2: Cyclooxygenase 2
MIB1: Mindbomb E3 Ubiquitin Protein Ligase 1
NF2: Neurofibromatosis type 2
TMA: Tissue microarray
VS: Vestibular schwannoma
